# ROR1 is a pseudokinase that is crucial for MET-driven tumorigenesis

**DOI:** 10.1186/1753-6561-7-S2-P43

**Published:** 2013-04-04

**Authors:** Luca Lazzari, Alessandra Gentile, Silvia Benvenuti, Livio Trusolino, Paolo M Comoglio

**Affiliations:** 1 Exploratory Research Laboratory, Institute for Cancer Research and Treatment (IRCC), Candiolo (TO), Italy; 2 Molecular Pharmacology Laboratory, Institute for Cancer Research and Treatment (IRCC), Candiolo (TO), Italy; 3 Department of Oncological Sciences, University of Turin Medical School, Candiolo (TO), Italy

## Background

Aberrant deregulation of some Receptor Tyrosine Kinases (RTKs) signalling underlies diverse facets of tumor pathobiology providing an attractive target for cancer therapy. Searching for novel cancer-associated RTKs is an important issue to discover new therapeutic opportunities. For this reason, we investigated the contribution of Receptor tyrosine kinase-like Orphan Receptor 1 (ROR1) to human cancer.

## Patients and methods

The level of ROR1 protein expression, phosphorylation and cellular growth response to RNAi-mediated ROR1 knockdown was evaluated by an integrated screening in a panel of 43 cancer cell lines. ROR1 auto-kinase activity and transphosphorylation were determined by biochemical assays. Functional consequences of ROR1 silencing were evaluated by several *in vitro* and *in vivo* biological assays.

## Results

We demonstrated that although ROR1 is expressed in approximately 75% of the screened cancer cell lines, only gastric carcinoma cells (HS746T) and non-small cell lung carcinoma cells (NCI-H1993) exhibit high levels of ROR1 tyrosine phosphorylation and experience growth inhibition upon ROR1 suppression. Biochemical assays revealed that ROR1 is a pseudokinase lacking autocatalytic activity. Intriguingly, the two phospho-ROR1 positive cell lines both exhibited amplification and constitutive activation of the *MET* oncogene. ROR1 phosphorylation was abrogated by MET inhibition, indicating MET dependent transphosphorylation of ROR1. Silencing of ROR1 in HS746T and NCI-H1993 cells impaired cellular proliferation, growth and migration *in vitro* and induced a dramatic inhibition of tumorigenesis *in vivo*.

## Conclusions

Our data show that ROR1 is a pseudokinase functionally transphosphorylated by MET RTK, suggesting a critical role for ROR1 in malignant phenotypes sustained by the *MET* oncogene.

## Financial support

AIRC special program Molecular Clinical Oncology “5x1000” and AIRC grants.

